# Frozen section is superior to imprint cytology for the intra-operative assessment of sentinel lymph node metastasis in Stage I Breast cancer patients

**DOI:** 10.1186/1477-7819-4-26

**Published:** 2006-05-17

**Authors:** Miki Mori, Keiichiro Tada, Motoko Ikenaga, Yumi Miyagi, Seiichiro Nishimura, Kaoru Takahashi, Masujiro Makita, Takuji Iwase, Fujio Kasumi, Mituru Koizumi

**Affiliations:** 1 Department of Breast Surgery, Cancer Institute Hospital, Tokyo, Japan; 2 Department of Cytology, Cancer Institute Hospital, Tokyo, Japan; 3 Department of Radiology, Cancer Institute Hospital, Tokyo, Japan

## Abstract

**Background:**

A standard intra-operative procedure for assessing sentinel lymph node metastasis in breast cancer patients has not yet been established.

**Patients and methods:**

One hundred and thirty-eight patients with stage I breast cancer who underwent sentinel node biopsy using both imprint cytology and frozen section were analyzed.

**Results:**

Seventeen of the 138 patients had sentinel node involvement. Results of imprint cytology included nine false negative cases (sensitivity, 47.1%). In contrast, only two cases of false negatives were found on frozen section (sensitivity, 88.2%). There were two false positive cases identified by imprint cytology (specificity, 98.3%). On the other hand, frozen section had 100% specificity.

**Conclusion:**

These findings suggest that frozen section is superior to imprint cytology for the intra-operative determination of sentinel lymph node metastasis in stage I breast cancer patients.

## Background

Sentinel node biopsy has become a common surgical procedure in the treatment of breast cancer. [1] This procedure can predict the presence of nodal metastasis with high accuracy and thereby prevent morbid axillary clearance in node negative breast cancer patients. This useful technique requires the intra-operative determination of metastasis in the sentinel nodes. If sentinel node metastasis is determined intra-operatively, axillary clearance can be performed at the same time, thus obviating the need for a second operation. However, it remains unclear what should be the standard technique for intra-operative sentinel lymph node determination. Two available procedures to determine the presence of nodal metastasis are frozen section and imprint cytology. Although a few studies have shown acceptable sensitivities for frozen section applied to intra-operative diagnosis, [2,3] many studies have claimed there are advantages to using imprint cytology in this setting. [4-8] In this study, we compare the usefulness of imprint cytology and frozen section in the intra-operative diagnosis of sentinel node metastasis. We also review and discuss the literature regarding these techniques.

## Patients and methods

From April 2000 to September 2003, we performed sentinel node mapping (SNM) in 138 breast cancer patients. Candidates for SNM had to meet pre-specified criteria, which included: a tumor size less than 2 cm; a node-negative tumor based on palpation and ultrasonography, and no evidence of distant metastasis.

The technique of sentinel node biopsy has been described previously. [9,10] Briefly, 1.5 mCi/ml of the radioactive tracer 99 mTc-rhenium sulphide or 99 mTc-phytate (Daiichi Radioisotope Laboratories, Ltd), was used. The radioactive agent was injected subdermally, close to the tumor. In all cases, a lymphoscintigraph was obtained one hour after injection. Additionally, a total of 5 ml of vital dye (indigocallumine) was injected intradermally and into the peri-tumoral space at the operation. We had an interval of more than five minutes between the injection and the incision. Sentinel lymph nodes were identified using a hand-held Gamma probe with the assistance of stained vessels and nodes.

Sentinel nodes were evaluated by both imprint cytology and frozen section. In general, removed sentinel nodes were divided into three sections. Furthermore, we tried to section the tissue such that the sectioned plane was parallel to the plane in which the maximal section area could be obtained. When the sentinel lymph nodes were too small for trisection, they were bisected. The sectioned surface of the sentinel node was imprinted onto the surface of a slide, which was immediately immersed into a 90% alcohol solution. The slides were then stained using the Papanicolaou method. They were examined by specialized cytologists and cytopathologists. Next, the sectioned sentinel lymph nodes were sent to the laboratory, and frozen sections were made with the use of Coldtome (Sakura Fine Technical Co. Ltd., Tokyo Japan). Each frozen section was cut from one level and stained with hematoxylin and eosin (H&E). The frozen sections were studied intra-operatively by a trained pathologist. The remaining tissue specimen from the frozen section was thawed, fixed in formalin, and embedded in paraffin. This permanent specimen, stained with H & E, was sectioned from one level and was examined by a pathologist postoperatively. The final diagnosis of lymph node metastasis was based on histopathological evaluation. No immunohistological staining technique was used routinely.

## Results

A total of 231 lymph nodes from 138 cases were evaluated by both imprint cytology and frozen section. Among these 138 patients, a total of 17 had axillary lymph node metastasis. The concordance between imprint cytology and the absolute diagnosis is shown in Table 1. According to imprint cytology there were nine false negative cases among 17 node positive patients (sensitivity, 47.1%). On the other hand, two false positive cases were identified by imprint cytology (specificity, 98.3%).

**Table 1:** Comparison between intraoperative imprint cytology and final histological diagnosis

	Node-positive cases at final diagnosis	Node-negative cases at final diagnosis	
	Node-positive cases at final diagnosis	Node-negative cases at final diagnosis	
Positive introperative imprint cytology	8	2	10
Negative introperative imprint cytology	9	119	128
	17	121	
	Sensitivity	47.1%	
	Specificity	98.3%	
	Positive Predictive Value	80.0%	
	Negative Predictive Value	93.0%	

The agreement between frozen section and final diagnosis is shown in Table 2. Frozen section detected lymph node metastasis in 15 of 17 node positive cases (sensitivity, 88.2%) and there were no false positive cases (specificity,100%).

**Table 2:** Comparison between intraoperative frozen section and final histological diagnosis

	Node-positive cases at final diagnosis	Node-negative cases at final diagnosis	
Positive in introperative frozen section	15	0	15
Negative in introperative frozen section	2	121	123
	17	121	
	Sensitivity	88.2%	
	Specificity	100%	
	Positive Predictive Value	100%	
	Negative Predictive Value	98.4%	

## Discussion

Our study demonstrates that frozen section is superior to imprint cytology for the intra-operative diagnosis of sentinel node metastasis. Frozen section had a higher sensitivity than imprint cytology, while its high specificity was comparable to that of imprint cytology.

Several reports in the literature have compared frozen section with imprint cytology, and most of these reports have recommended imprint cytology for intra-operative sentinel lymph node determination. [4,6,7] Imprint cytology is considered a rapid and convenient method with sensitivity and specificity similar to that obtained with frozen section. However, as seen in Table 3, which presents comparisons of sensitivities and specificities obtained from various reports, the sensitivity of imprint cytology is likely to be lower than that of frozen section. [6-8,11-20] Although some studies showed good sensitivities for imprint cytology, these investigators either did not investigate frozen sections [7,12] or they studied more sections using imprint cytology than using frozen section. [6]

**Table 3:** Reported results of intraoperative imprint cytology and frozen section

	imprint cytology		frozen section		number of cases
		
	sensitivity	specificity	sensitivity	specificity	
Present study	47.1	98.3	88.2	100	138
Leidenius et al. (2003)	68	99	83	99	375
Beach et al. (2003)	69	100	54	100	32
Liang et al. (2003)	62.5	100	62.5	100	20
Nagashima et al. (2003)	70.3	99.6	83.8	100	§ 303
Sauer et al. (2003)	58	100	77	100	§ 429
Motomura et al. (2000)	96	90.8	52	100	101
van Diest et al. (1999)	62	100	87	100	54
Fisher et al. (1993)	98	100	90.2	100	50

§ These numbers are based on the number of examnined lymph nodes

The characteristic feature of our study is that we compared imprint cytology and frozen section in an equal manner, because we studied the same sectioned surfaces using both methods. As a result, our data showed that the discrepancy in the sensitivities between these methods could be attributed mainly to sampling error associated with the imprint technique.

The evaluation of specificities in imprint cytology is also a complex issue. Generally, it is difficult to achieve a specificity of 100% using imprint cytology. There are at least two possible explanations for this difficulty. First, it is possible that benign specimens may be judged as containing malignant cells. In particular, the lobular type of breast cancer is believed to generate a false positive on imprint cytology due to the small and bland morphology of the cells. This means that some patients may undergo unnecessary axillary dissection. Unwarranted axillary clearance is clearly more problematic than a second operation for axillary dissection. The second reason is associated with micro-metastases. In cases of micro-metastases, it is possible that only imprint cytology can detect metastatic cells. However, when histopathological evaluation is required to define sentinel lymph node metastasis, the role of imprint cytology is limited.

For these reasons, we recommend frozen section rather than imprint cytology for the intra-operative diagnosis of sentinel lymph node metastasis.

We initially attempted to use imprint cytology as a modality complementary to frozen section for the intra-operative diagnosis of sentinel lymph node metastasis. Therefore, we used the Papanicolaou method for intra-operative cytological evaluation. Although this technique is time-consuming compared with other staining methods, we believe this procedure is greatly advantageous because it results in accurate evaluations.

The rate of positive sentinel node detection in patients with a sentinel node biopsy in our cohort is likely to be lower than that reported in other studies. The use of trisecting, which is nowadays inappropriate for the analysis of sentinel nodes, may be responsible for this result. However, we believe that ultrasonography, which was used in addition to physical examination to evaluate preoperative nodal status at our institution, contributed significantly to this consequence by allowing us to obtain more accurate nodal evaluations. The rate of positive sentinel node detection in patients who received a sentinel node biopsy should be low, because a high positive rate increases the possibility of missing axillary node metastases, both in sentinel and non-sentinel nodes. This, then, increases the likelihood of a subsequent salvage operation after swollen axillary lymph nodes become clinically apparent.

In the intra-operative diagnosis of lymph node metastasis, the management of micro-metastases is a difficult problem to solve. [8,18] Several new intra-operative approaches to micrometastases have been reported. [21,22] Their approaches are based on intra-operative thin sections, in which the nodes are examined thoroughly on the basis of frozen section. These time- and money-consuming methods are less likely to be introduced to routine clinical practice. The long-term prognostic impact of micro-metastasis should be established as soon as possible.

## Conclusion

This study demonstrates that frozen section is superior to imprint cytology for the intra-operative diagnosis of sentinel node metastasis with respect to both sensitivity and specificity. We currently recommend using frozen section rather than imprint cytology for detecting intra-operative lymph node metastasis.

## Competing interests

The author(s) declare that they have no competing interests.

## Authors' contributions

**KT **produced the study design, conducted literature searches and drafted the manuscript. **MM **analyzed all data and searched the literature. **MI **contributed to the cytological analysis. **SN**, **KT**, **MM**, **TI **and **FK **contributed to surgical interventions and collected patient data. **MK **contributed to the analysis of nuclear medicine and collected data. All authors read and approved the final manuscript
